# Cardiac wasting in patients with cancer

**DOI:** 10.1007/s00395-024-01079-5

**Published:** 2024-09-23

**Authors:** Markus S. Anker, Ahmed Mustafa Rashid, Javed Butler, Muhammad Shahzeb Khan

**Affiliations:** 1 German Centre for Cardiovascular Research (DZHK), Partner Site Berlin, Berlin, Germany; 2 Berlin Institute of Health Center for Regenerative Therapies (BCRT), Berlin, Germany; 3 Department of Cardiology, Angiology and Intensive Care CBF, Deutsches Herzzentrum Der Charité, Berlin, Germany; 4 Charité - Universitätsmedizin Berlin, Corporate Member of Freie Universität Berlin and Humboldt-Universität Zu Berlin, Campus Benjamin Franklin, Hindenburgdamm 30, 12200 Berlin, Germany; 5 Department of Medicine, Jinnah Sindh Medical University, Karachi, Pakistan; 6 Department of Medicine, University of Mississippi Medical Center, Jackson, MS USA; 7 Baylor Scott and White Research Institute, Baylor Scott and White Health, 3434 Live Oak Street, Dallas, TX 75204 USA; 8 Department of Cardiology, Baylor Scott and White Heart Hospital Plano, Plano, TX USA; 9 Department of Medicine, Baylor College of Medicine, Temple, TX USA

**Keywords:** Cardio-oncology, Cardiac wasting, Inflammation, Cardiac dysfunction

## Abstract

Patients with cancer face a significant risk of cardiovascular death, regardless of time since cancer diagnosis. Elderly patients are particularly more susceptible as cancer-associated cardiac complications present in advanced stage cancer. These patients may often present with symptoms observed in chronic heart failure (HF). Cardiac wasting, commonly observed in these patients, is a multifaceted syndrome characterized by systemic metabolic alterations and inflammatory processes that specifically affect cardiac function and structure. Experimental and clinical studies have demonstrated that cancer-associated cardiac wasting is linked with cardiac atrophy and altered cardiac morphology, which impairs cardiac function, particularly pertaining to the left ventricle. Therefore, this review aims to present a summary of epidemiologic data and pathophysiological mechanisms of cardiac wasting due to cancer, and future directions in this field.

## Introduction

Approximately 20–30% of patients with cancer die from cardiovascular-related causes, irrespective of the duration following their cancer diagnosis [32]. The life expectancy of patients with cancer has increased, leading to a high prevalence of complex symptoms and an aging population [33]. Elderly patients include an expanding cohort with an elevated susceptibility to cardiovascular risks due to the existence of common risk factors and shared disease processes of the underlying tumor [41]. More recent mortality trend analysis corroborates these findings, with a higher mortality attributed to heart failure (HF) in elderly patients with cancer (8.5% vs. 2.4% per year from 1999 to 2019) when compared to the younger patients [41]. Patients with advanced stages of cancer often present with reduced mobility, congestion, and breathlessness and have an increased risk of sudden death [2, 3]. Therefore, the importance of cardiac dysfunction as a leading factor in non-cancer-related mortality in these patients cannot be undermined. In advanced cancer, cachexia is commonly found in 30% to 80% of patients, varying based on the specific cancer type, stage, and existing comorbidities [51]. Unintentional weight loss and muscle atrophy distinguish cachexia in advanced cancer [38]. Cancer may coexist with other symptoms like fatigue, reduced fitness, and shortness of breath [2]. These presentations are also observed in chronic HF. Despite cardiac atrophy and impaired functionality observed in 40% of patients with cancer and most experimental models, this cohort remains one of the most understudied subtypes [57]. Therefore, this review aims to present a summary of epidemiologic data and the pathophysiological mechanisms of cardiac wasting due to cancer, as well as future directions in this field.

## Epidemiologic evidence of *cancer*-related cardiac wasting

### Preclinical evidence

**Fig. 1:**
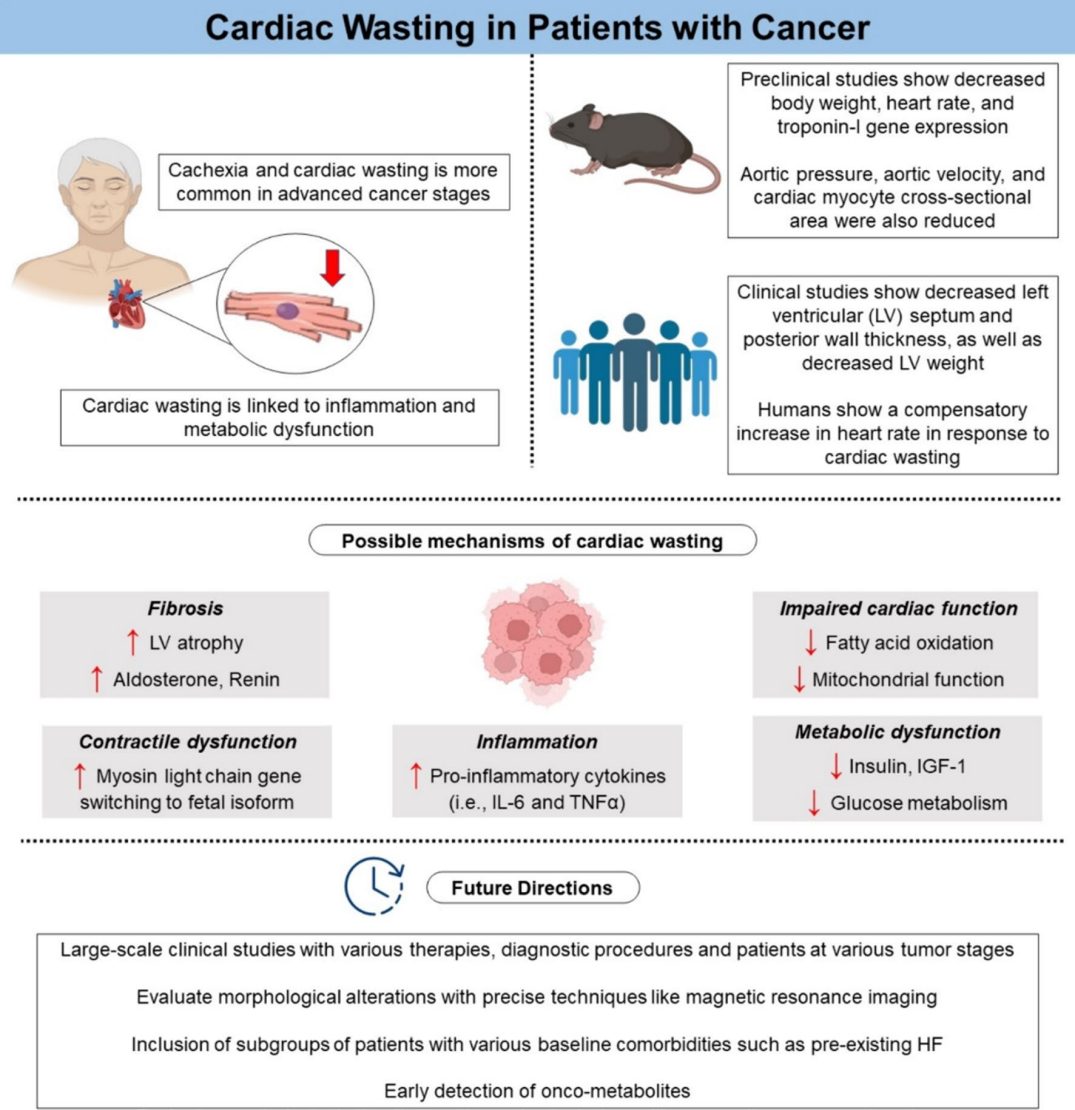
Cardiac wasting in patients with cancer

**Fig. 2:**
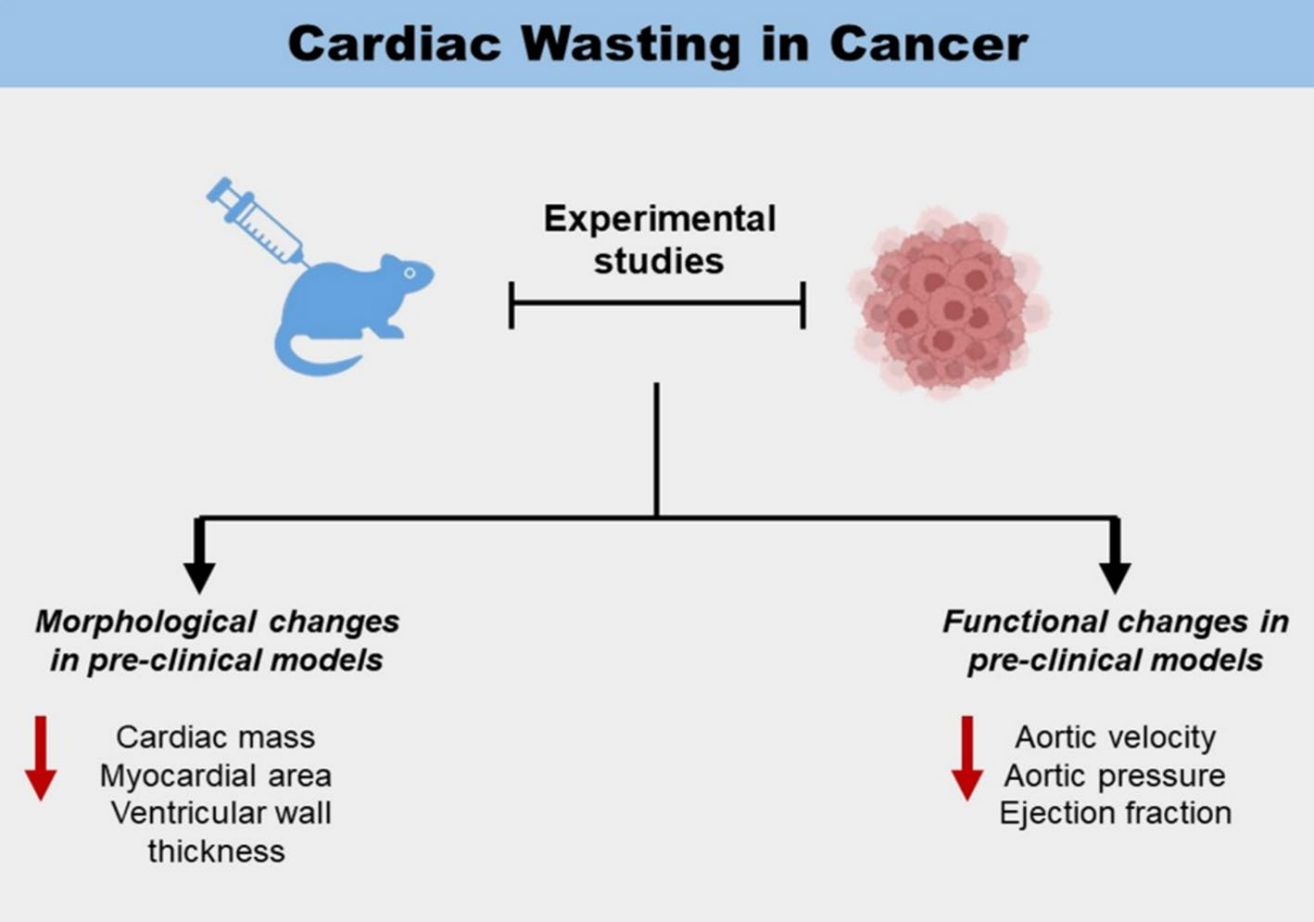
Summary of findings from experimental studies

Evaluation of physiologic changes in cancer has been elucidated by preclinical experimental studies [10, 56, 63] (Fig. 1). In the first study of cancer in mice, cachexia due to cancer led to a 23% reduction in body weight, a 21% reduction in heart rate, and a 38% reduction in troponin-I gene expression between the tumor and non-tumor groups [56]. Quantitative real-time polymerase chain reaction indicated heightened levels of inflammation with substantial upregulation of transcript levels of interleukin (IL), where notably IL-6 and IL-6-receptors increased by 5.7-fold and 2.3-fold, respectively [56]. Moreover, correlations with structural changes, such as fibrosis and altered cardiomyocyte ultrastructure, were observed, aligning with previous studies demonstrating reduced cardiac muscle mass and a depletion of contractile and myofibrillar proteins in tumor-bearing mice (Fig. 2) [14, 31].

Cardiac atrophy with increased autophagy, reduced cardiac myocyte size, and fewer sarcomere proteins has been observed in a preclinical mouse model [10]. In addition, a 31% reduction in cardiac myocyte cross-sectional area due to cancer was observed, which aligns with a prior tumor model, which showed a 50% reduction in left ventricular (LV) axon length [10, 34]. Moreover, structural atrophy was coexistent with functional deteriorations of 30% and 16% reductions in aortic pressure and aortic velocity [10]. Despite cardiac atrophy, LV ejection fraction (EF) was preserved in a mouse model, consistent with findings in other models of cardiac atrophy [4, 10, 61]. Cardiac atrophy can be potentially reversed by inhibiting certain receptors involved in muscle atrophy. Activin type 2 receptor (ActRIIB) is a high-affinity receptor that mediates signaling by certain transforming growth factor-β (TGF-β) family ligands, including myostatin and activin. ActRIIB pathway antagonism can potentially reverse muscle atrophy [63]. Particularly, antagonism of ActRIIB in mouse models nullified the effects of muscle-wasting ubiquitin ligases and reduced muscle-protein ubiquitination [63].

### Clinical evidence

In humans, two retrospective studies shaped the initial evidence on cardiac wasting due to cancer [5, 49]. Autopsy analysis of 26 patients who died from cancer indicated fibrotic remodeling in the cardiac muscle irrespective of cachexia status and showed a 26% reduction in cardiac mass, a 50% reduction in LV mass, a corresponding 30% reduction in lean mass, and a 12% reduction in LV-wall thickness due to cancer [49]. In another study, autopsy data from 177 cancer patients, including 54 patients with cachexia, also revealed a 19% reduction in cardiac mass due to cancer without any change in left and right ventricular diameters in comparison to patients without cachexia [5]. The presence of cancer was also linked with cardiac dysfunction and reduced LVEF [49]. Moreover, serum aldosterone, renin, and brain natriuretic peptide (BNP) levels were elevated in patients with cachexia by 2.1-fold, 2.9-fold, and 3.0-fold, respectively [49]. Hence, these findings reinforce the intricate link between cancer-related cachexia and cardiac wasting, with profound implications for disease symptoms, clinical well-being, and patient survival (Fig. 1).

Initial prospective clinical data from 50 patients with non-small cell lung cancer aimed to assess outcomes pre- and post-chemotherapy treatment [25]. Echocardiographic analysis showed evidence of cardiac wasting in patients with non-small cell lung cancer, with a 9% reduction in LV mass after 4 months of carboplatin‐based palliative chemotherapy. Patients with the greatest LV mass reductions had a significantly greater decline in global longitudinal strain (GLS) and QRS duration [25]. However, these structural changes did not correlate with changes in LVEF, consistent with observations in mouse models [4, 10, 61]. These findings corroborate existing evidence that healthy adults had less reductions in LV mass [21, 23]. However, Kazemi-Bajestani et al. did not assess the functional status of patients comprehensively. The study was also inadequately powered to demonstrate prognostic assessments due to a small sample size and a limited follow-up of 3.7 months. This is noteworthy, as the survival time for patients with metastatic non-small cell lung cancer is approximately 15 months. Hence, it is plausible that investigators only observed a fraction of LV atrophy.

Recent clinical data provide adequately powered prospective evidence. Data from 300 advanced cancer patients without pre-existing significant cardiovascular disease exhibited cardiac wasting across diverse cancer types, irrespective of their specific anticancer therapy status, including those receiving naive, non-cardiotoxic, or cardiotoxic treatments [29]. On echocardiography, LV mass was significantly reduced by 25% and 28% in cancer patients, with and without cachexia, respectively. The presence of cachexia was also associated with structural changes, including reduced LV, left atrial and right atrial volumes, thinner myocardial walls, and reduced posterior wall thickness [12, 29]. These structural alterations were linked to notable declines in functional performance parameters, including 6-min walking distance, stairclimbing power, maximum handgrip strength, and peak oxygen consumption [12, 29]. However, Lena et al. [29] did not stratify patients by treatment-naïve status or treatment history, creating uncertainty as to whether the observed changes were solely attributable to the underlying cancer or were influenced by cardiotoxic therapies.

In existing clinical studies including patients with cancer, cardiac wasting was assessed by various parameters, with the most common being LVEF, followed by cardiac or LV mass, LV-wall thickness, and levels of BNP, aldosterone, or renin [5, 23, 25, 49]. However, LVEF being a functional assessment may not accurately reflect cardiac wasting, especially in the early stages where compensatory mechanisms may keep EF normal [40]. Hence, cardiovascular magnetic resonance and high-resolution echocardiography can provide an accurate diagnosis of LV mass and chamber dimensions. Further, levels of inflammatory markers and biomarkers, such as creatine kinase and BNP, can further aid in diagnosing underlying cardiac atrophy.

## Possible mechanisms of *cancer*-related cardiac wasting

Existing literature extensively reviews cardiotoxicity induced by anticancer drugs and targeted therapies but inadequately addresses the pathogenesis of cardiac atrophy directly caused by cancer itself.

### Metabolic dysfunction and inflammation

The pathophysiology of cancer cachexia centers on metabolic dysfunction, marked by an imbalance of high catabolism and low anabolism [64]. Several factors contribute to metabolic dysfunction during cancer cachexia, including the obstructive effects of local tumors, competition by tumor cells for nutrients, and a skewed energy balance in healthy tissues exacerbated by the rapid and uncontrolled growth of tumor cells [1, 16, 48]. This metabolic disruption has been evident in preclinical studies using C26 tumor models in mice, where shifts in cardiac metabolism reflect a whole‐body metabolic imbalance. Tian et al. demonstrated altered cardiac gene expression in tumor-bearing mice, including increased levels of BNP, reduced activity of peroxisome proliferator-activated receptor alpha (PPARα), and a shift in myosin heavy chain (MHC) isoforms from an “adult” to an “embryonic” phenotype, indicative of cardiac remodeling [55, 56].

The metabolic alterations include a significant shift toward catabolism, increased inflammation, and a decrease in protein synthesis. In the realm of cancer cachexia, IL-6 and tumor necrosis factor- α (TNFα) are notable pro-inflammatory cytokines. Elevated levels of IL-6 could potentially provoke alterations in cardiac function by reducing fatty-acid oxidation and impairing mitochondrial function [45, 52]. In addition, both IL-6 and TNFα are involved in regulating muscle-wasting pathways, enhancing energy expenditure by promoting inflammatory processes, and inhibiting protein synthesis [48, 59]. For example, in various rodent models of cancer cachexia, these cytokines have been consistently found to be elevated in the heart, directly impacting cardiac function [39, 43]. These alterations result in a reduction of cardiac mass, characterized by thinning of the ventricular walls and subsequent atrophy, which adversely affects cardiac functional parameters.

**Fig. 3:**
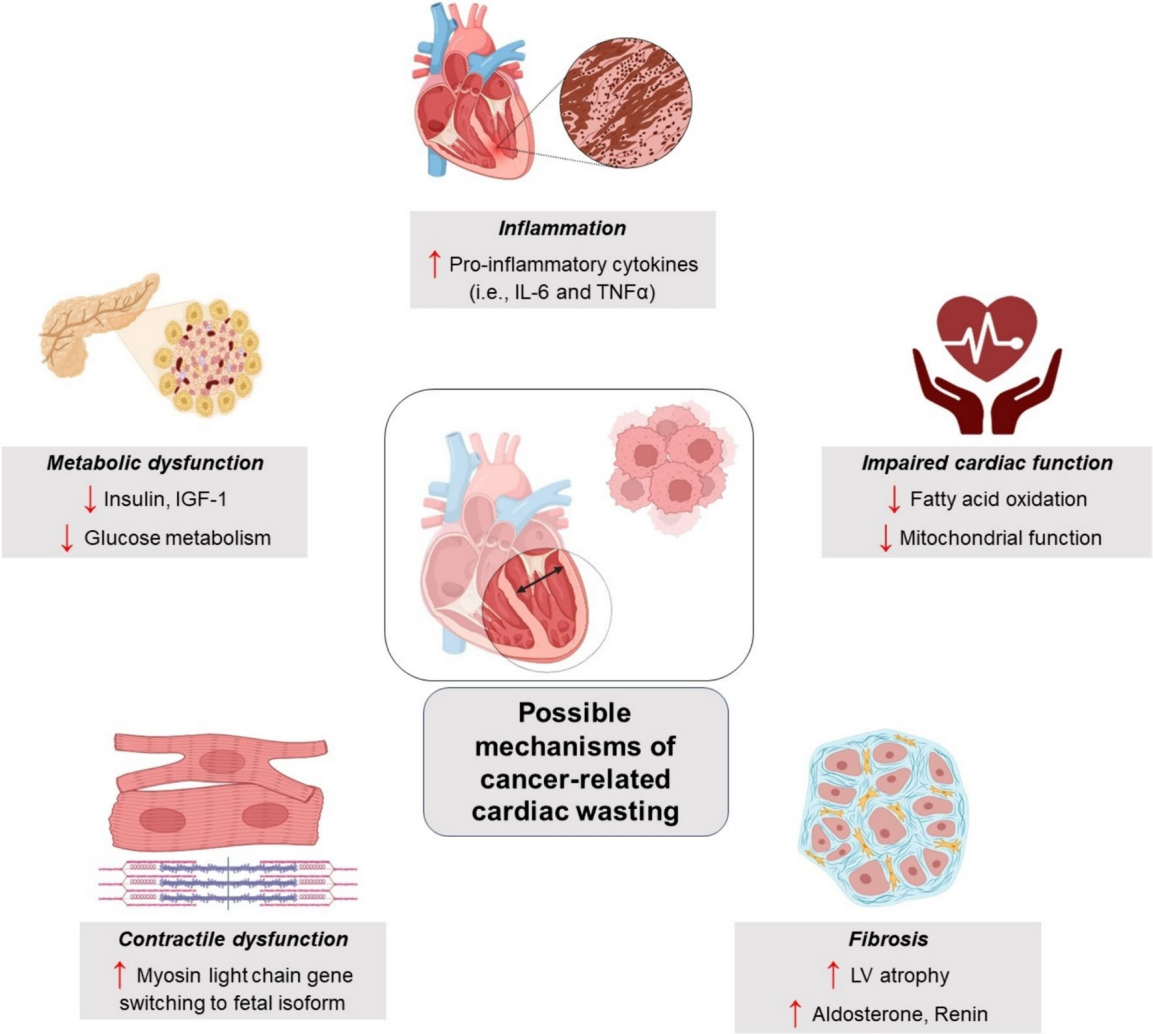
Possible mechanisms of cancer-related cardiac wasting

Both malignant and cardiac cells release factors that disrupt normal metabolic functions, such as insulin and insulin-like growth factor 1 (IGF-1). Insufficient insulin levels have been observed in numerous patients experiencing cachexia, as well as in rodents with advanced tumors [11, 54]. This depletion of insulin in malignancy leads to abnormal glucose metabolism, causing reduced uptake of glucose in cardiac tissue and subsequent cardiac atrophy. Hence, cancer-induced metabolic derangements lead to cardiac wasting, MHC alterations, and cardiac dysfunction (Fig. 3).

Metabolic dysfunction also heightens systemic inflammation, thereby influencing the progression of tumor growth [8, 44]. This tumor growth contributes more significantly to cardiac dysfunction than cancer cachexia, as observed in a preclinical study [13]. Recently, in addition to IL-6, the involvement of nuclear factor-κB (NFκB) has been associated with cardiac atrophy induced by cancer cachexia [46, 62]. NFκB is crucial for FAS-ligand-induced apoptosis, and heightened circulating levels of the endogenous NFkB-activator, osteoprotegerin, are linked with a higher risk of mortality, particularly in cardiovascular disease [30].

### Impaired contractile function

Metabolic deterioration and a trend toward catabolism, along with a rise in inflammation, can lead to cardiac dysfunction in patients with cancer and in cachectic animals. The prevailing cardiac dysfunction frequently observed in both clinical and experimental research represents cardiac atrophy and alterations in cardiac morphology, which lead to poor cardiac performance, particularly pertaining to the LV [56, 63]. Alterations may arise from NFκB, widespread inflammation, and cancer growth, subsequently activating ubiquitin ligases, which are a part of ubiquitin proteasome system-related catabolism [25, 35].

Cardiac wasting also includes derangements in contractile function due to gene switching in myocardial cells, where MHC shifts from ‘adult’ to ‘fetal’ variants [18, 42, 60]. Adult forms of MHC exhibit lower ATPase activity in comparison to MHC-α, which tends to be more prevalent during the embryonic developmental stage and demands comparatively higher energy (Fig. 3). This isoform switch is linked with higher glucose consumption, lower fatty-acid oxidation, systolic dysfunction, and an overall worsening of cardiac function [36, 57, 63]. Moreover, in a proteomic study of a C26 tumor mouse model, MHC isoform switching was associated with destabilized sarcomeres in cardiac myocytes [47]. This destabilization results in the release of sarcomeric proteins like desmin for degradation, a process to overcome the metabolic stress on the cardiovascular system due to tumor burden [47]. While MHC alterations may represent a compensatory action of the cardiac muscle to conserve energy, they simultaneously impair contractile performance [10, 56]. Impaired cardiac function can manifest fatigue symptoms, resulting in reduced physical activity and exercise and consequently aggravating cardiac cachexia. Eventually, a detrimental cycle is initiated, leading to higher morbidity in cancer cachexia.

Fibrosis and LV atrophy can potentially occur due to elevated aldosterone levels (Fig. 3). Data from untreated mice and patients with cancer shows a potential link between increased fibrosis and elevated aldosterone levels [9, 49]. Moreover, tumor-bearing mice showed a reduction in aldosterone levels and fibrosis after treatment with spironolactone [49]. The release of aldosterone appears to be partially driven by the renin–angiotensin–aldosterone system, as an increase in renin circulating levels in both mice and humans has been detected [49]. Yet, notably, there can likewise be RAS-independent processes involved, as higher cortisol levels were also observed with reported adrenal gland hypertrophy in experimental rats [49]. Moreover, elevated plasma concentrations of fibrinogen, tissue inhibitor of metalloproteinases-1, and monocyte chemoattractant protein-1 in mouse models point toward structural cardiac anomalies and inflammation, further enhancing the effects of aldosterone [58].

## Other diseases with cardiac wasting

Patients with cancer cachexia may experience tiredness, dyspnea, and poor exercise tolerance, which are well-recognized symptoms of chronic HF [24]. However, cardiac wasting in patients with HF and cancer has remained underrepresented in existing literature and has garnered limited focus in both clinical practice and research thus far. The American Society of Echocardiography and the European Association of Cardiovascular Imaging have consistently observed that the majority of extensive population-based studies use LV mass indexed for body surface area (BSA), which effectively incorporates the patient’s height and weight [27, 29]. In advanced cancer, patients undergo a reduction in total body weight; hence, a corresponding drop in BSA leads to either *A)* an increased ratio of LV mass to BSA (if LV mass remains unchanged) or *B)* an unchanged ratio of LV mass to BSA (if LV mass and BSA both decline simultaneously). This adjustment of LV mass for BSA potentially obscures the absolute magnitude of reductions in LV mass [29]. Hence, cardiac wasting was previously documented only in cases of chronic HF when widespread cachexia, characterized by total weight loss, had already occurred [17].

In patients with cancer, impaired cardiac function reduces cardiac output. Cachexia leads to LV-wall thinning, which elevates ventricular wall stress, according to Laplace’s law, and increases the susceptibility to HF under hemodynamic overload [22]. Preclinical models also indicate higher cardiac fibrosis in patients with cachexia, which subsequently leads to myocardial stiffness [26, 49]. Furthermore, inflammation and metabolic derangements can lead to myofibrillar protein dysfunction due to gene switching in myocardial cells, where MHC shifts from ‘adult’ to ‘fetal’ variants and disrupts efficient myocardial relaxation and filling. Moreover, inflammation and metabolic derangement further worsen cardiac atrophy, including myocardial thinning and fibrosis. Hence, these underlying mechanisms progress to cardiac dysfunction, leading to a HF-like syndrome.

Severe acute malnutrition exists in edematous (kwashiorkor) or nonedematous (marasmic) forms in children. Malnutrition has also shown cardiac dysfunction in clinical studies. Echocardiography in patients with kwashiorkor revealed a smaller heart due to diminished myocardial tissue [7]. This is consistent with recent findings, where patients with kwashiorkor had a reduced LV outflow tract diameter and poor systolic function (stroke volume and cardiac output) [53]. Moreover, diastolic blood pressure and systemic vascular resistance were also found to be elevated in experimental mice with weaning protein deficiency [6].

## Treatment for cardiac wasting in preclinical models of *cancer*

### Pharmacologic therapies

Within the context of cancer-induced cardiac cachexia, the utilization of HF therapies such as angiotensin-converting enzyme inhibitors (ACEi), angiotensin receptor blockers (ARB), mineralocorticoid receptor antagonists (MRA), and beta blockers has displayed varying efficacy in preclinical models [20, 44, 59]. ACEi, in particular imidapril, did not improve survival in a preclinical model [49]. However, ACEi can reduce cardiac load, cardiac dysfunction, and the progression of cardiac wasting in tumor-bearing mice [44, 59]. These outcomes are mainly driven by reduced endothelial dysfunction, modifications in IGF-1 levels, and lower circulating levels of inflammatory markers such as IL‐6 and TNF-α. These findings are also consistent with ARBs, albeit evidence originating from preclinical data only. In C26 tumor-bearing mice, losartan prevented the reductions in fractional shortening, EF, stroke volume, and posterior wall thickness, while also mitigating increase in LV end-diastolic diameter [50].

MRAs and beta blockers have also shown positive results in tumor models. Spironolactone has shown better survival, maintained LV mass, and improved systolic function by improving LVEF and fractional shortening in AH-130 tumor-bearing mice [49]. Similarly, in AH-130 tumor-bearing mice, bisoprolol increased overall survival, increased heart weight, and reduced the decline in LV mass and end-diastolic diameter and volume, but did not improve systolic function [49]. The impact of therapeutic agents on cardiac function aligns with signaling pathways regulating the balance between catabolic and anabolic processes in cardiac tissue. Bisoprolol and spironolactone normalized cell growth, proliferation, and survival-associated protein kinases, while imidapril did not [49]. However, there is lack of specific studies in human patients evaluating medications to counteract cardiac wasting. Therefore, rigorous clinical investigations are warranted in patients with cancer to assess the efficacy of different HF therapies for cardiac wasting.

### Exercise

A practical and effective non-pharmacologic therapy in the general population is aerobic endurance or resistance training. Aerobic training can possibly improve levels of anti-inflammatory cytokines, including IL-10, to modify insulin signaling and alleviate effects of metabolic derangements on the heart [1]. High- and moderate-intensity exercise has shown potential for partially restoring LVEF, mitigate remodeling, and augment LV mass in preclinical models [15, 19, 37]. This may improve exercise tolerance, fatigue, life expectancy, and quality of life [28]. However, these observations are mainly derived from preclinical studies in tumor mice models, as there is a scarcity of clinical evidence regarding exercise intervention in patients with cancer and cardiac wasting. Therefore, clinical studies are warranted to assess whether pharmacologic therapy and/or exercise training can improve outcomes in patients with cancer and cardiac wasting.

## Future directions

The majority of evidence exists from experimental studies or small-scale clinical studies. In animal models, the presence of oncometabolites increases the risk of cardiac dysfunction [24]. However, these oncometabolites are often not reported in clinical studies until the advanced phases of the tumor, where patients are often already undergoing palliative care. Hence, poor early detection can further deteriorate the quality of life for these patients. Large-scale, comprehensive clinical studies can bridge the gap in real-world clinical evidence by incorporating various therapies and diagnostic procedures and including patients at various stages of tumor development. These studies should also incorporate more precise techniques of evaluation, such as magnetic resonance imaging, to evaluate morphologic alterations with a simultaneous comparison of various treatments. Patients with various baseline comorbidities should be included in subgroups, as certain comorbidities, such as pre-existing HF, could increase the risk of cardiac wasting in patients with cancer [25]. Furthermore, these studies can categorize patients based on their treatment-naïve status and exposure to cardiotoxic therapies to better assess the direct effects of cancer on cardiac wasting. Moreover, these large-scale studies can target limitations in existing literature. In Barkhudaryan et al., patients with cachexia received better long-term cancer treatment compared to patients without cachexia [5]. This possibly led to the early death of patients in the non-cachexia group due to the late diagnosis of cancer; hence, counterintuitively, patients with cachexia can sometimes also present with better survival.

## Conclusion

Cancer-associated cardiac wasting can involve various organs, widespread inflammation, and impaired contractile function. Existing literature demonstrate that cardiac structural and functional changes can lead to symptoms that may present as HF-like syndrome. However, despite its evident clinical importance, no therapies exist to address cardiac wasting in patients with cancer. Therefore, the current pathophysiological and therapeutic evidence is mainly derived from experimental studies. Large-scale studies are required to further assess the impact of various therapeutic approaches on cardiac wasting. Moreover, future studies should also investigate effects of different cancer types on cardiac wasting and early diagnosis.

## References

[1] Allan J, Buss LA, Draper N, Currie MJ (2022) Exercise in people with cancer: a spotlight on energy regulation and cachexia. Front Physiol 13:836804. 10.3389/fphys.2022.836804 35283780 10.3389/fphys.2022.836804 PMC8914107

[2] Anker MS, Hadzibegovic S, Lena A, Belenkov Y, Bergler-Klein J, de Boer RA, Farmakis D, von Haehling S, Iakobishvili Z, Maack C, Pudil R, Skouri H, Cohen-Solal A, Tocchetti CG, Coats AJS, Seferović PM, Lyon AR, Heart Failure Association Cardio-Oncology Study Group of the European Society of Cardiology (2019) Recent advances in cardio-oncology: a report from the 'Heart Failure Association 2019 and World Congress on Acute Heart Failure 2019'. ESC Heart Fail 6:1140–1148 10.1002/ehf2.12551 10.1002/ehf2.12551 PMC6989292 31884717

[3] Anker MS, von Haehling S, Landmesser U, Coats AJS, Anker SD (2018) Cancer and heart failure-more than meets the eye: common risk factors and co-morbidities. Eur J Heart Fail 20:1382–1384. 10.1002/ejhf.1252 29943887 10.1002/ejhf.1252

[4] Artaza JN, Reisz-Porszasz S, Dow JS, Kloner RA, Tsao J, Bhasin S, Gonzalez-Cadavid NF (2007) Alterations in myostatin expression are associated with changes in cardiac left ventricular mass but not ejection fraction in the mouse. J Endocrinol 194:63–76. 10.1677/JOE-07-0072 17592022 10.1677/JOE-07-0072

[5] Barkhudaryan A, Scherbakov N, Springer J, Doehner W (2017) Cardiac muscle wasting in individuals with cancer cachexia. ESC Heart Fail 4:458–467. 10.1002/ehf2.12184 29154433 10.1002/ehf2.12184 PMC5695173

[6] de Belchior AC, Angeli JK, Faria Tde O, Siman FD, Silveira EA, Meira EF, da Costa CP, Vassallo DV, Padilha AS (2012) Post-weaning protein malnutrition increases blood pressure and induces endothelial dysfunctions in rats. PLoS ONE 7:e34876. 10.1371/journal.pone.0034876 22529948 10.1371/journal.pone.0034876 PMC3329540

[7] Bergman JW, Human DG, De Moor MM, Schulz JM (1988) Effect of kwashiorkor on the cardiovascular system. Arch Dis Child 63:1359–1362. 10.1136/adc.63.11.1359 3202643 10.1136/adc.63.11.1359 PMC1779151

[8] Bordignon C, Dos Santos BS, Rosa DD (2022) Impact of cancer cachexia on cardiac and skeletal muscle: role of exercise training. Cancers (Basel) 14:342. 10.3390/cancers14020342 35053505 10.3390/cancers14020342 PMC8773522

[9] Briet M, Schiffrin EL (2010) Aldosterone: effects on the kidney and cardiovascular system. Nat Rev Nephrol 6:261–273. 10.1038/nrneph.2010.30 20234356 10.1038/nrneph.2010.30

[10] Cosper PF, Leinwand LA (2011) Cancer causes cardiac atrophy and autophagy in a sexually dimorphic manner. Cancer Res 71:1710–1720. 10.1158/0008-5472.CAN-10-3145 21163868 10.1158/0008-5472.CAN-10-3145 PMC3049989

[11] Coss CC, Clinton SK, Phelps MA (2018) Cachectic cancer patients: immune to checkpoint inhibitor therapy? Clin Cancer Res 24:5787–5789. 10.1158/1078-0432.CCR-18-1847 30018117 10.1158/1078-0432.CCR-18-1847 PMC6279566

[12] Cramer L, Hildebrandt B, Kung T, Wichmann K, Springer J, Doehner W, Sandek A, Valentova M, Stojakovic T, Scharnagl H, Riess H, Anker SD, von Haehling S (2014) Cardiovascular function and predictors of exercise capacity in patients with colorectal cancer. J Am Coll Cardiol 64:1310–1319. 10.1016/j.jacc.2014.07.948 25257631 10.1016/j.jacc.2014.07.948

[13] Dostal C, Szabo L, Aioanei C, Abraham D, Zins K, Bakiri L, Wagner E, Podesser BK, Kiss A (2022) Dissecting the progression of cardiac dysfunction in tumor-bearing mice. Cardiovasc Res 118(cvac066):247. 10.1093/cvr/cvac066.247

[14] Drott C, Lönnroth C, Lundholm K (1989) Protein synthesis, myosin ATPase activity and myofibrillar protein composition in hearts from tumour-bearing rats and mice. Biochem J 264:191–198. 10.1042/bj2640191 2481444 10.1042/bj2640191 PMC1133563

[15] Fernandes LG, Tobias GC, Paixão AO, Dourado PM, Voltarelli VA, Brum PC (2020) Exercise training delays cardiac remodeling in a mouse model of cancer cachexia. Life Sci 260:118392. 10.1016/j.lfs.2020.118392 32898523 10.1016/j.lfs.2020.118392

[16] Finke D, Heckmann MB, Frey N, Lehmann LH (2021) Cancer—a major cardiac comorbidity with implications on cardiovascular metabolism. Front Physiol 12:729713. 10.3389/fphys.2021.729713 34899373 10.3389/fphys.2021.729713 PMC8662519

[17] Florea VG, Moon J, Pennell DJ, Doehner W, Coats AJ, Anker SD (2004) Wasting of the left ventricle in patients with cardiac cachexia: a cardiovascular magnetic resonance study. Int J Cardiol 97:15–20. 10.1016/j.ijcard.2003.05.050 15336800 10.1016/j.ijcard.2003.05.050

[18] Gidh-Jain M, Huang B, Jain P, Gick G, El-Sherif N (1998) Alterations in cardiac gene expression during ventricular remodeling following experimental myocardial infarction. J Mol Cell Cardiol 30:627–637. 10.1006/jmcc.1997.0628 9515038 10.1006/jmcc.1997.0628

[19] Hardee JP, Counts BR, Carson JA (2017) Understanding the role of exercise in cancer cachexia therapy. Am J Lifestyle Med 13:46–60. 10.1177/1559827617725283 30627079 10.1177/1559827617725283 PMC6311610

[20] Hweidi IM, Al-Omari AK, Rababa MJ, Al-Obeisat SM, Hayajneh AA (2021) Cardiac cachexia among patients with chronic heart failure: a systematic review. Nurs Forum 56:916–924. 10.1111/nuf.12623 34091923 10.1111/nuf.12623

[21] Jordan JH, Castellino SM, Meléndez GC, Klepin HD, Ellis LR, Lamar Z, Vasu S, Kitzman DW, Ntim WO, Brubaker PH, Reichek N, D’Agostino RB Jr, Hundley WG (2018) Left ventricular mass change after anthracycline chemotherapy. Circ Heart Fail 11:e004560. 10.1161/CIRCHEARTFAILURE.117.004560 29991488 10.1161/CIRCHEARTFAILURE.117.004560 PMC6729136

[22] Kadowaki H, Akazawa H, Shindo A, Ueda T, Ishida J, Komuro I (2024) Shared and reciprocal mechanisms between heart failure and cancer—an emerging concept of heart-cancer axis. Circ J 88:182–188. 10.1253/circj.CJ-23-0838 38092383 10.1253/circj.CJ-23-0838

[23] Karimian S, Stein J, Bauer B, Teupe C (2016) Impact of severe obesity and weight loss on systolic left ventricular function and morphology: assessment by 2-dimensional speckle-tracking echocardiography. J Obes 2016:2732613. 10.1155/2016/2732613 27006823 10.1155/2016/2732613 PMC4781964

[24] Karlstaedt A, Zhang X, Vitrac H, Harmancey R, Vasquez H, Wang JH, Goodell MA, Taegtmeyer H (2016) Oncometabolite d-2-hydroxyglutarate impairs α-ketoglutarate dehydrogenase and contractile function in rodent heart. Proc Natl Acad Sci U S A 113:10436–10441. 10.1073/pnas.1601650113 27582470 10.1073/pnas.1601650113 PMC5027422

[25] Kazemi-Bajestani SMR, Becher H, Butts C, Basappa NS, Smylie M, Joy AA, Sangha R, Gallivan A, Kavsak P, Chu Q, Baracos VE (2019) Rapid atrophy of cardiac left ventricular mass in patients with non-small cell carcinoma of the lung. J Cachexia Sarcopenia Muscle 10:1070–1082. 10.1002/jcsm.12451 31293070 10.1002/jcsm.12451 PMC6818459

[26] Kong P, Christia P, Frangogiannis NG (2014) The pathogenesis of cardiac fibrosis. Cell Mol Life Sci 71:549–574. 10.1007/s00018-013-1349-6 23649149 10.1007/s00018-013-1349-6 PMC3769482

[27] Lang RM, Badano LP, Mor-Avi V, Afilalo J, Armstrong A, Ernande L, Flachskampf FA, Foster E, Goldstein SA, Kuznetsova T, Lancellotti P, Muraru D, Picard MH, Rietzschel ER, Rudski L, Spencer KT, Tsang W, Voigt JU (2015) Recommendations for cardiac chamber quantification by echocardiography in adults: an update from the American Society of Echocardiography and the European Association of Cardiovascular Imaging. J Am Soc Echocardiogr 28:1-39.e14. 10.1016/j.echo.2014.10.003 25559473 10.1016/j.echo.2014.10.003

[28] Lee IM, Shiroma EJ, Lobelo F, Puska P, Blair SN, Katzmarzyk PT, Lancet Physical Activity Series Working Group (2012) Effect of physical inactivity on major non-communicable diseases worldwide: an analysis of burden of disease and life expectancy. Lancet 380:219–229 10.1016/S0140-6736(12)61031-9 10.1016/S0140-6736(12)61031-9 PMC3645500 22818936

[29] Lena A, Wilkenshoff U, Hadzibegovic S, Porthun J, Rösnick L, Fröhlich AK, Zeller T, Karakas M, Keller U, Ahn J, Bullinger L, Riess H, Rosen SD, Lyon AR, Lüscher TF, Totzeck M, Rassaf T, Burkhoff D, Mehra MR, Bax JJ, Butler J, Edelmann F, Haverkamp W, Anker SD, Packer M, Coats AJS, von Haehling S, Landmesser U, Anker MS (2023) Clinical and prognostic relevance of cardiac wasting in patients with advanced cancer. J Am Coll Cardiol 81:1569–1586. 10.1016/j.jacc.2023.02.039 37076211 10.1016/j.jacc.2023.02.039

[30] Lieb W, Gona P, Larson MG, Massaro JM, Lipinska I, Keaney JF Jr, Rong J, Corey D, Hoffmann U, Fox CS, Vasan RS, Benjamin EJ, O’Donnell CJ, Kathiresan S (2010) Biomarkers of the osteoprotegerin pathway: clinical correlates, subclinical disease, incident cardiovascular disease, and mortality. Arterioscler Thromb Vasc Biol 30:1849–1854. 10.1161/ATVBAHA.109.199661 20448212 10.1161/ATVBAHA.109.199661 PMC3039214

[31] Lundholm K, Edström S, Ekman L, Karlberg I, Bylund AC, Scherstén T (1978) A comparative study of the influence of malignant tumor on host metabolism in mice and man: evaluation of an experimental model. Cancer 42:453–461. 10.1002/1097-0142(197808)42:2%3c453::aid-cncr2820420212%3e3.0.co;2-t 679148 10.1002/1097-0142(197808)42:2<453::aid-cncr2820420212>3.0.co;2-t

[32] Mansouri I, Allodji RS, Hill C, El-Fayech C, Pein F, Diallo S, Schwartz B, Vu-Bezin G, Veres C, Souchard V, Dumas A, Bolle S, Thomas-Teinturier C, Pacquement H, Munzer M, Bondiau PY, Berchery D, Fresneau B, Oberlin O, Diallo I, De Vathaire F, Haddy N (2019) The role of irradiated heart and left ventricular volumes in heart failure occurrence after childhood cancer. Eur J Heart Fail 21:509–518. 10.1002/ejhf.1376 30592114 10.1002/ejhf.1376

[33] Miller KD, Nogueira L, Mariotto AB, Rowland JH, Yabroff KR, Alfano CM, Jemal A, Kramer JL, Siegel RL (2019) Cancer treatment and survivorship statistics. CA Cancer J Clin 69:363–385. 10.3322/caac.21565 31184787 10.3322/caac.21565

[34] Mühlfeld C, Das SK, Heinzel FR, Schmidt A, Post H, Schauer S, Papadakis T, Kummer W, Hoefler G (2011) Cancer induces cardiomyocyte remodeling and hypoinnervation in the left ventricle of the mouse heart. PLoS ONE 6:e20424. 10.1371/journal.pone.0020424 21637823 10.1371/journal.pone.0020424 PMC3102720

[35] Murphy KT (2016) The pathogenesis and treatment of cardiac atrophy in cancer cachexia. Am J Physiol Heart Circ Physiol 310:H466-477. 10.1152/ajpheart.00720.2015 26718971 10.1152/ajpheart.00720.2015

[36] Nakao K, Minobe W, Roden R, Bristow MR, Leinwand LA (1997) Myosin heavy chain gene expression in human heart failure. J Clin Invest 100:2362–2370. 10.1172/JCI119776 9410916 10.1172/JCI119776 PMC508434

[37] Parry TL, Hayward R (2018) Exercise protects against cancer-induced cardiac cachexia. Med Sci Sports Exerc 50:1169–1176. 10.1249/MSS.0000000000001544 29315166 10.1249/MSS.0000000000001544

[38] Peixoto da Silva S, Santos JMO, Silva CE, MP, Gil da Costa RM, Medeiros R, (2020) Cancer cachexia and its pathophysiology: links with sarcopenia, anorexia and asthenia. J Cachexia Sarcopenia Muscle 11:619–635. 10.1002/jcsm.12528 32142217 10.1002/jcsm.12528 PMC7296264

[39] Peyta L, Jarnouen K, Pinault M, Coulouarn C, Guimaraes C, Goupille C, de Barros JP, Chevalier S, Dumas JF, Maillot F, Hatch GM, Loyer P, Servais S (2015) Regulation of hepatic cardiolipin metabolism by TNFα: implication in cancer cachexia. Biochim Biophys Acta 1851:1490–1500. 10.1016/j.bbalip.2015.08.008 26327596 10.1016/j.bbalip.2015.08.008

[40] Potter E, Marwick TH (2018) Assessment of left ventricular function by echocardiography: the case for routinely adding global longitudinal strain to ejection fraction. JACC Cardiovasc Imaging 11:260–274. 10.1016/j.jcmg.2017.11.017 29413646 10.1016/j.jcmg.2017.11.017

[41] Raisi-Estabragh Z, Kobo O, Freeman P, Petersen SE, Kolman L, Miller RJH, Roguin A, Van Spall HGC, Vuong J, Yang EH, Mamas MA (2022) Temporal trends in disease-specific causes of cardiovascular mortality amongst patients with cancer in the USA between 1999 and 2019. Eur Heart J Qual Care Clin Outcomes 9:54–63. 10.1093/ehjqcco/qcac016 35435219 10.1093/ehjqcco/qcac016 PMC9745666

[42] Razeghi P, Young ME, Alcorn JL, Moravec CS, Frazier OH, Taegtmeyer H (2001) Metabolic gene expression in fetal and failing human heart. Circulation 104:2923–2931. 10.1161/hc4901.100526 11739307 10.1161/hc4901.100526

[43] Rupert JE, Narasimhan A, Jengelley DHA, Jiang Y, Liu J, Au E, Silverman LM, Sandusky G, Bonetto A, Cao S, Lu X, O’Connell TM, Liu Y, Koniaris LG, Zimmers TA (2021) Tumor-derived IL-6 and trans-signaling among tumor, fat, and muscle mediate pancreatic cancer cachexia. J Exp Med 218:e20190450. 10.1084/jem.20190450 33851955 10.1084/jem.20190450 PMC8185651

[44] Saha S, Singh PK, Roy P, Kakar SS (2022) Cardiac Cachexia: Unaddressed Aspect in Cancer Patients. Cells 11:990. 10.3390/cells11060990 35326441 10.3390/cells11060990 PMC8947289

[45] Saito S, Aikawa R, Shiojima I, Nagai R, Yazaki Y, Komuro I (1999) Endothelin-1 induces expression of fetal genes through the interleukin-6 family of cytokines in cardiac myocytes. FEBS Lett 456:103–107. 10.1016/s0014-5793(99)00936-9 10452539 10.1016/s0014-5793(99)00936-9

[46] Shadfar S, Couch ME, McKinney KA, Weinstein LJ, Yin X, Rodríguez JE, Guttridge DC, Willis M (2011) Oral resveratrol therapy inhibits cancer-induced skeletal muscle and cardiac atrophy in vivo. Nutr Cancer 63:749–762. 10.1080/01635581.2011.563032 21660860 10.1080/01635581.2011.563032 PMC3623008

[47] Shum AMY, Poljak A, Bentley NL, Turner N, Tan TC, Polly P (2018) Proteomic profiling of skeletal and cardiac muscle in cancer cachexia: alterations in sarcomeric and mitochondrial protein expression. Oncotarget 9:22001–22022. 10.18632/oncotarget.25146 29774118 10.18632/oncotarget.25146 PMC5955146

[48] Soto ME, Pérez-Torres I, Rubio-Ruiz ME, Manzano-Pech L, Guarner-Lans V (2022) Interconnection between cardiac cachexia and heart failure-protective role of cardiac obesity. Cells 11:1039. 10.3390/cells11061039 35326490 10.3390/cells11061039 PMC8946995

[49] Springer J, Tschirner A, Haghikia A, von Haehling S, Lal H, Grzesiak A, Kaschina E, Palus S, Pötsch M, von Websky K, Hocher B, Latouche C, Jaisser F, Morawietz L, Coats AJ, Beadle J, Argiles JM, Thum T, Földes G, Doehner W, Hilfiker-Kleiner D, Force T, Anker SD (2014) Prevention of liver cancer cachexia-induced cardiac wasting and heart failure. Eur Heart J 35:932–941. 10.1093/eurheartj/eht302 23990596 10.1093/eurheartj/eht302 PMC3977133

[50] Stevens SC, Velten M, Youtz DJ, Clark Y, Jing R, Reiser PJ, Bicer S, Devine RD, McCarthy DO, Wold LE (2015) Losartan treatment attenuates tumor-induced myocardial dysfunction. J Mol Cell Cardiol 85:37–47. 10.1016/j.yjmcc.2015.05.007 25988231 10.1016/j.yjmcc.2015.05.007 PMC4530048

[51] Sun L, Quan XQ, Yu S (2015) An epidemiological survey of cachexia in advanced cancer patients and analysis on its diagnostic and treatment status. Nutr Cancer 67:1056–1062. 10.1080/01635581.2015.1073753 26317149 10.1080/01635581.2015.1073753

[52] Tanaka T, Kanda T, Takahashi T, Saegusa S, Moriya J, Kurabayashi M (2004) Interleukin-6-induced reciprocal expression of SERCA and natriuretic peptides mRNA in cultured rat ventricular myocytes. J Int Med Res 32:57–61. 10.1177/147323000403200109 14997707 10.1177/147323000403200109

[53] Tennant IA, Barnett AT, Thompson DS, Kips J, Boyne MS, Chung EE, Chung AP, Osmond C, Hanson MA, Gluckman PD, Segers P, Cruickshank JK, Forrester TE (2014) Impaired cardiovascular structure and function in adult survivors of severe acute malnutrition. Hypertension 64:664–671. 10.1161/HYPERTENSIONAHA.114.03230 24980666 10.1161/HYPERTENSIONAHA.114.03230

[54] Thackeray JT, Pietzsch S, Stapel B, Ricke-Hoch M, Lee CW, Bankstahl JP, Scherr M, Heineke J, Scharf G, Haghikia A, Bengel FM, Hilfiker-Kleiner D (2017) Insulin supplementation attenuates cancer-induced cardiomyopathy and slows tumor disease progression. JCI Insight 2(10):e93098. 10.1172/jci.insight.93098 28515362 10.1172/jci.insight.93098 PMC5436547

[55] Tian M, Asp ML, Nishijima Y, Belury MA (2011) Evidence for cardiac atrophic remodeling in cancer-induced cachexia in mice. Int J Oncol 39:1321–1326. 10.3892/ijo.2011.1150 21822537 10.3892/ijo.2011.1150

[56] Tian M, Nishijima Y, Asp ML, Stout MB, Reiser PJ, Belury MA (2010) Cardiac alterations in cancer-induced cachexia in mice. Int J Oncol 37:347–353. 10.3892/ijo_00000683 20596662 10.3892/ijo_00000683

[57] Tichy L, Parry TL (2023) The pathophysiology of cancer-mediated cardiac cachexia and novel treatment strategies: a narrative review. Cancer Med 12:17706–17717. 10.1002/cam4.6388 37654192 10.1002/cam4.6388 PMC10524052

[58] Vanhoutte D, Schellings M, Pinto Y, Heymans S (2006) Relevance of matrix metalloproteinases and their inhibitors after myocardial infarction: a temporal and spatial window. Cardiovasc Res 69:604–613. 10.1016/j.cardiores.2005.10.002 16360129 10.1016/j.cardiores.2005.10.002

[59] Vudatha V, Devarakonda T, Liu C, Freudenberger DC, Riner AN, Herremans KM, Trevino JG (2022) Review of mechanisms and treatment of cancer-induced cardiac cachexia. Cells 11:1040. 10.3390/cells11061040 35326491 10.3390/cells11061040 PMC8947347

[60] Wellner M, Dechend R, Park JK, Shagdarsuren E, Al-Saadi N, Kirsch T, Gratze P, Schneider W, Meiners S, Fiebeler A, Haller H, Luft FC, Muller DN (2005) Cardiac gene expression profile in rats with terminal heart failure and cachexia. Physiol Genomics 20:256–267. 10.1152/physiolgenomics.00165.2004 15623567 10.1152/physiolgenomics.00165.2004

[61] Welsh DC, Dipla K, McNulty PH, Mu A, Ojamaa KM, Klein I, Houser SR, Margulies KB (2001) Preserved contractile function despite atrophic remodeling in unloaded rat hearts. Am J Physiol Heart Circ Physiol 281:H1131-1136. 10.1152/ajpheart.2001.281.3.H1131 11514279 10.1152/ajpheart.2001.281.3.H1131

[62] Wysong A, Couch M, Shadfar S, Li L, Rodriguez JE, Asher S, Yin X, Gore M, Baldwin A, Patterson C, Willis MS (2011) NF-κB inhibition protects against tumor-induced cardiac atrophy in vivo. Am J Pathol 178:1059–1068. 10.1016/j.ajpath.2010.12.009 21356358 10.1016/j.ajpath.2010.12.009 PMC3070568

[63] Zhou X, Wang JL, Lu J, Song Y, Kwak KS, Jiao Q, Rosenfeld R, Chen Q, Boone T, Simonet WS, Lacey DL, Goldberg AL, Han HQ (2010) Reversal of cancer cachexia and muscle wasting by ActRIIB antagonism leads to prolonged survival. Cell 142:531–543. 10.1016/j.cell.2010.07.011 20723755 10.1016/j.cell.2010.07.011

[64] Ziemons J, Smidt ML, Damink SO, Rensen SS (2021) Gut microbiota and metabolic aspects of cancer cachexia. Best Pract Res Clin Endocrinol Metab 35:101508. 10.1016/j.beem.2021.101508 33648847 10.1016/j.beem.2021.101508

